# A Novel Function for the Conserved Glutamate Residue in the Walker B Motif of Replication Factor C

**DOI:** 10.3390/genes4020134

**Published:** 2013-03-26

**Authors:** Ankita Chiraniya, Jeff Finkelstein, Mike O’Donnell, Linda B. Bloom

**Affiliations:** 1 Department of Biochemistry and Molecular Biology, University of Florida, Gainesville, FL 32610, USA; E-Mail: ankitachiraniya@gmail.com; 2 Howard Hughes Medical Institute, The Rockefeller University, New York, NY 10021, USA; E-Mails: finkelstein_jeff@hotmail.com (J.F.); Odonnel@mail.rockefeller.edu (M.O.)

**Keywords:** DNA replication, clamp loader, AAA+ ATPase, Walker B motif, sliding clamp, ATP hydrolysis

## Abstract

In all domains of life, sliding clamps tether DNA polymerases to DNA to increase the processivity of synthesis. Clamp loaders load clamps onto DNA in a multi-step process that requires ATP binding and hydrolysis. Like other AAA+ proteins, clamp loaders contain conserved Walker A and Walker B sequence motifs, which participate in ATP binding and hydrolysis, respectively. Mutation of the glutamate residue in Walker B motifs (or DExx-boxes) in AAA+ proteins typically reduces ATP hydrolysis by as much as a couple orders of magnitude, but has no effect on ATP binding. Here, the Walker B Glu in each of the four active ATP sites of the eukaryotic clamp loader, RFC, was mutated to Gln and Ala separately, and ATP binding- and hydrolysis-dependent activities of the quadruple mutant clamp loaders were characterized. Fluorescence-based assays were used to measure individual reaction steps required for clamp loading including clamp binding, clamp opening, DNA binding and ATP hydrolysis. Our results show that the Walker B mutations affect ATP-binding-dependent interactions of RFC with the clamp and DNA in addition to reducing ligand-dependent ATP hydrolysis activity. Here, we show that the Walker B glutamate is required for ATP-dependent ligand binding activity, a previously unknown function for this conserved Glu residue in RFC.

## 1. Introduction

DNA replication requires the coordinated activity of many enzymes and proteins. Members of the AAA+ (ATPases associated with a variety of cellular activities) family of ATPases contribute to many steps in DNA replication including recognizing and unwinding origins where DNA synthesis will be initiated, unwinding duplex DNA to give DNA polymerases access to the template, and loading sliding clamps onto DNA (reviewed in [1,2]). Sliding clamps are ring-shaped proteins that encircle DNA and at the same time bind to DNA polymerases to increase the processivity of DNA synthesis by decreasing polymerase dissociation from DNA [3,4]. Without sliding clamps, synthesis by DNA polymerases would be too inefficient, due to frequent dissociation from the template, to support genome duplication.

Sliding clamps are assembled onto DNA by the activity of clamp loaders, which are AAA+ family members. This clamp loading reaction requires clamp loaders to bind clamps, open clamps, place open clamps around DNA, close clamps, and release the clamps to allow binding of DNA polymerases. This mechanical reaction is driven by ligand-induced conformational changes in the clamp loader. ATP binding promotes binding of the clamp loader to the clamp and DNA [5,6,7,8,9]. DNA binding triggers hydrolysis of ATP, and ATP hydrolysis stimulates release of the clamp on DNA [5,10,11].

**Figure 1:**
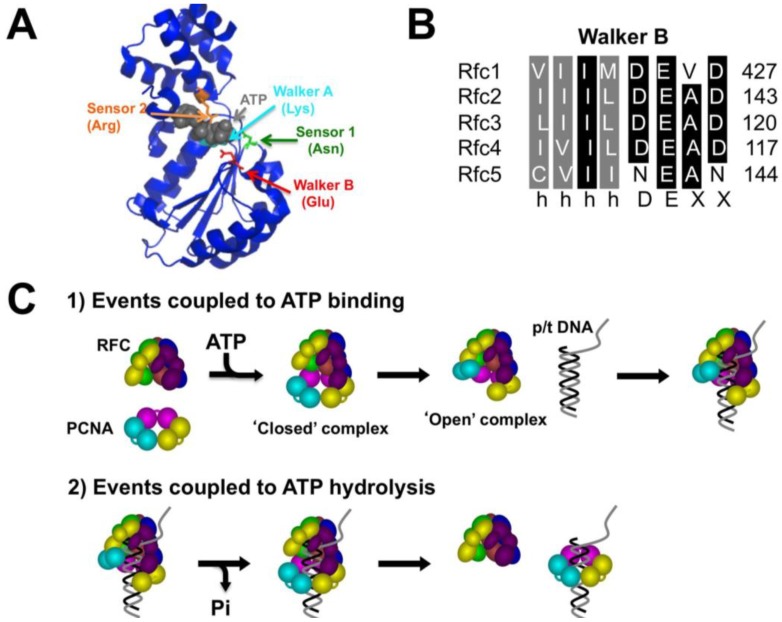
Structural features of replication factor C (RFC) and its interaction with ATP. (**a**) Ribbon diagram of the *S. cerevisiae* Rfc4 subunit bound to ATPγS (gray spheres) with conserved ATP site residues including the Walker A Lys (cyan), Walker B Glu (red), Sensor 1 Asn (green) and sensor 2 Arg (orange) shown as sticks. (**b**) Walker B sequence motifs in each of the RFC subunits. (**c**) The clamp loading reaction can be divided into two phases based on ATP requirements: (1) formation of a ternary clamp loader clamp DNA complex promoted by ATP binding, and (2) release of the clamp on DNA promoted by ATP hydrolysis. The diagram illustrates a structural model for RFC and PCNA based on crystal structures of clamp loaders and clamps [18,19,30,31]. Individual protein domains represented by spheres or ovals; note that Rfc1 contains an extra C-terminal domain that lies in the gap between Rfc1 and Rfc5.

As typical of AAA+ family members, clamp loaders are oligomeric assemblies of subunits that contain a conserved protein fold, the AAA+ module (reviewed in [12]). In addition to the AAA+ module, clamp loader subunits contain unique *C*-terminal domains that interact with one another to form a pentameric ring. The AAA+ modules are attached to the *C*-terminal domains to form a more open structure such that the clamp loader resembles a cap. Each AAA+ module contains two domains, and ATP binds at a site on the first domain and is sandwiched between the two domains (Figure 1A). Conserved nucleotide interacting sequence motifs including the Walker A and B motifs and a polar sensor 1 residue are located in the first domain and the basic sensor 2 residue is located in the second [13,14,15,16,17]. In AAA+ oligomers, ATP sites are also located at the interface of two subunits such that residues from the adjacent subunit including a conserved Arg finger form an integral part of the ATP active site. Given the location of ATP binding sites between two domains in a single subunit and at the interface of domains in a complex [14,15,18,19], mechanisms by which ATP binding and hydrolysis could promote conformational changes within individual subunits and within the complex as a whole are easy to envision.

The clamp loading reaction can be divided into two stages based on the ATP requirements (Figure 1C). In the first stage, ATP binding promotes clamp binding and opening as well as DNA binding. In the second stage, DNA binding promotes ATP hydrolysis that in turn stimulates clamp and DNA release. Key residues in the Walker A and B motifs are believed to be important for nucleotide binding and hydrolysis, respectively [20,21,22]. The Walker B motif contains an acidic amino acid residue, usually Asp, that is believed to coordinate a Mg^2+^ ion to promote ATP hydrolysis. AAA+ proteins contain a second conserved acidic residue that is usually Glu, and the consensus sequence is hhhhDExx in which h is a hydrophobic residue and xx residues are not well conserved but often AD [16]. This DExx-box was first recognized in helicases [23,24,25]. In P-loop NTPases that lack this second acidic residue in the Walker B motif, a Glu residue is usually found in the same spatial location relative to ATP [26,27]. These Glu residues have been proposed to activate a water molecule to hydrolyze ATP [22,25,27]. Thus, mutation of the Walker B Glu residue would be predicted to decrease the ATP hydrolysis activity of clamp loaders and hydrolysis-dependent steps such as release of the clamp on DNA, and this has been shown for an archaeal clamp loader [28]. However, recent work with another ATPase, bacterial SecA, has shown that mutation of the conserved glutamate to glutamine affects ATP-dependent conformational transitions and dynamics [29]. Similarly, in this work, we show that the Walker B Glu residue in replication factor C (RFC) contributes to ATP-dependent binding to PCNA and DNA.

## 2. Results and Discussion

### 2.1. Generation of RFC Walker B Mutants

Clamp loaders are well-characterized members of the AAA+ family proteins. RFC contains five subunits and each subunit contains sequences that are conserved among AAA+ proteins including the Walker B motif or DExx-box. To determine whether the Walker B Glu residues in RFC are important to ATP hydrolysis only, or whether they also make other contributions to enzyme activity, the activities of RFC Walker B (WB) mutants were measured for different steps of the clamp loading reaction. The conserved glutamate (E) residue in the WB motif was mutated to glutamine (Q) or alanine (A) to generate two types of WB mutants, referred to as WB-EQ or WB-EA mutants, respectively. Mutations were made in the four active ATPase subunits, Rfc1 (Glu-425), Rfc2 (Glu-141), Rfc3 (Glu-118), and Rfc4 (Glu-115) to generate a quadruple RFC mutant. Rfc5 naturally contains a mutation in the WB motif (D141N) and lacks a trans-acting arginine finger both of which reduce its ATPase activity. The mutant complexes were expressed and purified in the same way as WT RFC. Effects of the WB-EQ and WB-EA mutations on individual steps in the clamp loading reaction were evaluated.

The *N*-terminal region, the first 283 amino acids, of Rfc1 has DNA binding activity [32,33]. Deletion of these residues does not adversely affect clamp loading activity, and in some assays, actually simulates activity possibly by reducing nonspecific DNA binding [34,35,36]. Nor does deletion of this region affect cell viability or DNA replication [34]. In all RFC complexes used in this study, this *N*-terminal region was replaced with a 6X His-tag and kinase recognition motif [37,38], and the RFC complex lacking mutations in the ATP site is referred to as wild-type RFC.

### 2.2. Measurement of PCNA Binding and Opening

ATP binding to RFC promotes PCNA binding and opening, and ATP hydrolysis is not required for either of these interactions [5,39,40,41]. The nonhydrolyzable ATP analog, ATPγS, supports both PCNA binding and opening by RFC [5,41]. Therefore, if the only function of WB Glu residues is in catalyzing ATP hydrolysis, then mutation of these glutamates should not affect either PCNA binding or opening.

#### 2.2.1. PCNA Binding

**Figure 2:**
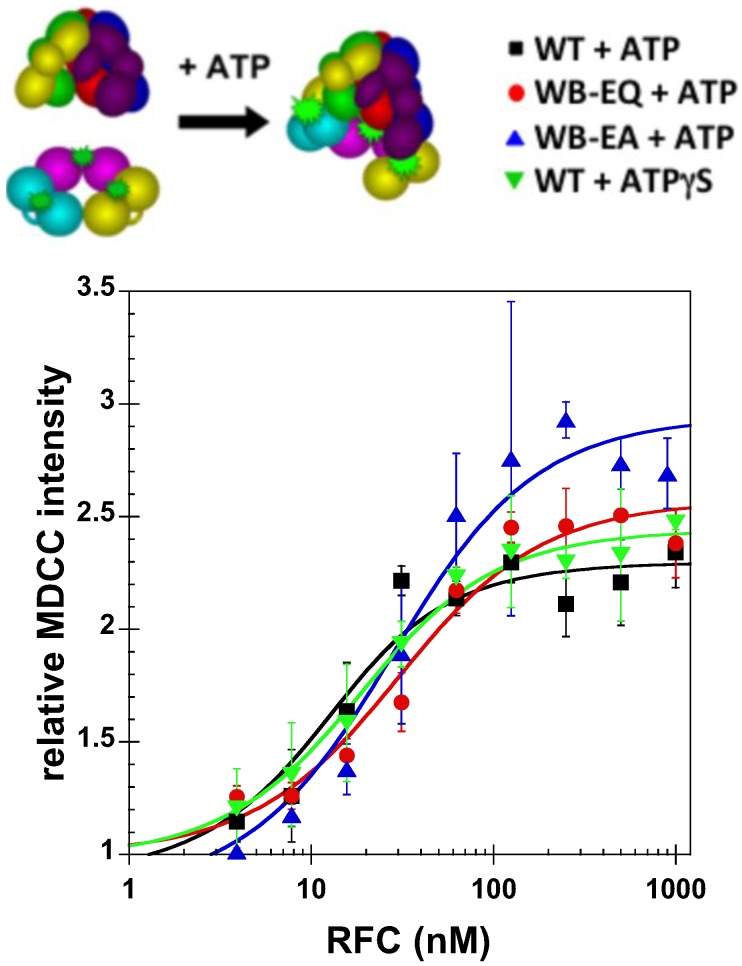
Clamp binding activities of WT RFC and Walker B mutants. Binding of WT RFC to PCNA-MDCC was measured in the presence of ATP (black squares) or ATPγS (green inverted triangles), and binding of WB-EQ (red circles) and WB-EA mutants (blue triangles) to PCNA-MDCC was measured in the presence of ATP. The relative intensity of MDCC at 470 nm is plotted as a function of RFC concentration for solutions containing 10 nM PCNA-MDCC and 0.5 mM ATP or ATPγS. Data shown are the average of three independent experiments. Error bars represent standard deviation.

A fluorescence intensity-based binding assay [42] was used to measure equilibrium binding of RFC to PCNA. The fluorescence of 10 nM MDCC labeled PCNA (PCNA-MDCC) was measured in presence of 0.5 mM ATP and increasing concentrations of RFC. Binding of RFC to PCNA-MDCC increases the fluorescence of MDCC in a concentration-dependent manner (Figure 2). A dissociation constant, *K_d_*, of 7.1 ± 2.1 nM was calculated for WT RFC in presence of ATP, which is comparable to the *K_d_* value of 1.3 nM measured previously using surface plasmon resonance [5]. *K_d_* values were higher for both WB mutants binding PCNA-MDCC. Calculated *K_d_* values were 25 ± 1 nM and 27 ± 12 nM, for the WB-EQ and WB-EA mutants, respectively.

Given that PCNA binding should not require ATP hydrolysis, weaker PCNA binding by the WB mutants should not be due to a defect in ATP hydrolysis. For comparison, we measured the effect of the “non-hydrolyzable” analog of ATP, ATPγS (5'-O-(3-thio)triphosphate) on PCNA binding activity of WT RFC [6]. A *K_d_* value of 13 ± 2 nM was calculated from PCNA-MDCC binding data in assays with 0.5 mM ATPγS. Hence, ATPγS increased the *K_d_* value by about a factor of 2.

#### 2.2.2. PCNA Opening

**Figure 3:**
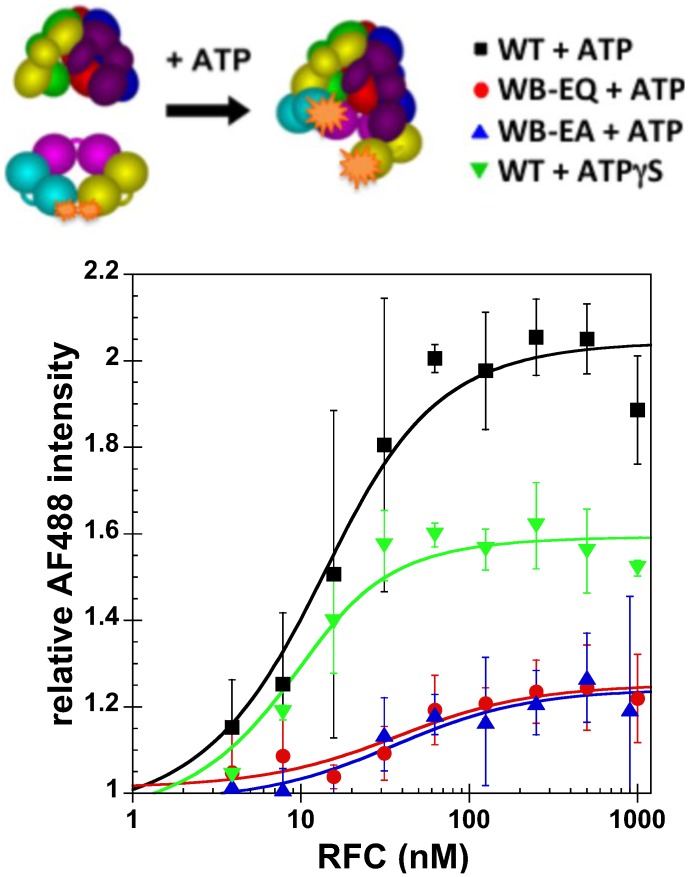
Clamp opening activities of WT RFC and Walker B mutants. Binding of WT RFC to PCNA-AF488 was measured in the presence of ATP (black squares) or ATPγS (green inverted triangles), and binding of the WB-EQ mutant (red circles) and WB-EA mutant (blue triangles) to PCNA-AF488 was measured in the presence of ATP. The relative intensity of AF488 at 517 nm is plotted as a function of RFC concentration for solutions containing 10 nM PCNA-AF488 and 0.5 mM ATP or ATPγS. Data shown are the average of three independent experiments. Error bars represent standard deviation.

As with PCNA binding, PCNA opening requires ATP binding but not hydrolysis [40,41], and therefore, if the WB Glu residues contribute only to ATP hydrolysis, PCNA opening should not be affected. PCNA opening was measured using a fluorescence “unquenching” assay [43]. In this assay, PCNA is labeled with Alexa Fluor 488 (AF488), one molecule on each side of the monomer interfaces. When PCNA is closed, the two fluorophores interact and are quenched. When PCNA is opened at one interface, two fluorophores are separated and the quenching is relieved. The fluorescence of 10 nM AF488-labeled PCNA (PCNA-AF488_2_) was measured in presence of 0.5 mM ATP and increasing concentrations of RFC (Figure 3). Two important types of information can be obtained from this assay. The RFC-concentration-dependence of the increase in fluorescence provides a measure of equilibrium binding constants, and the relative values of AF488 intensity at saturating RFC concentrations reflect the fraction of PCNA molecules in an open conformation. When PCNA-AF488_2_ was titrated with WT RFC, the fluorescence intensity increased by a factor of about 2 at saturating concentrations of RFC. In contrast, the titration of PCNA-AF488_2_ with the WB mutants gave only about a 1.2-fold increase in AF488 fluorescence. These data indicate that at equilibrium WT RFC forms about 5 times more open RFC•PCNA complexes than are formed with either of the WB mutant RFC complexes. The PCNA opening assay also shows that WB mutants have a lower affinity for PCNA than does WT RFC as observed in the PCNA-MDCC binding assay (Figure 2). A dissociation constant, *K_d_*, of 11 ± 4 nM was calculated for the WT RFC in presence of ATP. The *K_d_* values observed for the mutants were 40 ± 27 nM (WB-EQ) and 40 ± 13 nM (WB-EA). The error in the *K_d_* value calculated from PCNA opening data is greater than from binding data because the overall change in fluorescence was relatively small for the mutants.

To determine whether the decreased opening activity for the mutants is due to decreased ATP hydrolysis, PCNA opening by WT RFC was measured in assays with ATPγS. In these experiments, 0.5 mM ATPγS was substituted for ATP. When PCNA-AF488_2_ was titrated with WT RFC in assays with ATPγS, a smaller increase in AF488 fluorescence (1.5-fold) was observed at saturating RFC concentrations than in assays with ATP, but the *K_d_* value (5.8 ± 2.6 nM) was the same. The effect of ATPγS on PCNA opening differs from the effects of the WB mutations. The WB mutations affected both the fraction of PCNA molecules opened and the *K_d_* value, whereas ATPγS only affects the fraction of PCNA molecules opened, and the opening defect is smaller than for the WB mutants.

ATP binding to RFC induces conformational changes that increase the affinity of RFC for PCNA and stabilizes PCNA in an open conformation. Given that the affinity of the WB mutants for PCNA and the fraction of PCNA molecules that are opened by the WB mutants are both decreased relative to WT RFC, this would suggest that the WB mutations affect ATP-dependent conformational changes required for these interactions. One possibility is that interactions between the conserved Glu and ATP are important for ATP-binding induced conformational changes in RFC. A second possibility is that mutations to the Glu residue reduce ATP binding to RFC and as a result affect ATP-dependent interactions with PCNA. 

### 2.3. Measurement of ATP Binding

**Figure 4:**
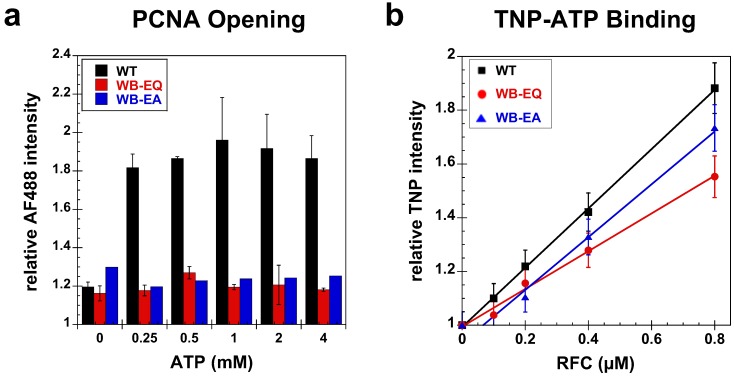
ATP binding to WT RFC and WB mutants measured by PCNA opening and TNP-ATP fluorescence. (**a**) Binding of WT RFC (black), the WB-EQ RFC mutant (red) and the WB-EA RFC mutant (blue) to PCNA-AF488 was measured in assays using the ATP concentrations indicated. The relative intensity of AF488 at 517 nm is plotted as a function of ATP concentration for solutions containing 10 nM PCNA-AF488 and 250 nM each RFC. Data shown are the average of three independent experiments (WT and WB-EQ mutant). Error bars represent standard deviation. The average two independent experiments is shown for the WB-EA mutant. (**b**) Binding of TNP-ATP to WT RFC and RFC WB mutants was measured by measuring the increase in TNP fluorescence that occurs on binding to RFC. The relative fluorescence of TNP was measured in assays containing 50 µM TNP-ATP and increasing concentrations of RFC as indicated. Data shown are the average of three independent experiments with error bars representing the standard deviation.

To test the possibility that decreased PCNA opening (and binding) by the WB mutants is due to weaker ATP binding, opening of PCNA-AF488_2_ was measured as a function of ATP concentration. The rationale being that if ATP binding to the WB mutants is weaker, increased ATP concentrations will increase ATP binding. The fluorescence of 10 nM PCNA-AF488 was measured in presence of 250 nM RFC and 0–4 mM ATP (Figure 4a). The fraction of clamps in an open conformation does not change for WT RFC over a concentration range of 0.25 to 4 mM ATP as indicated by intensity values that are the same, 1.8 to 2, within experimental error. For WT RFC, this result is consistent with reported *K_d_* values of less than 2 µM for ATP binding [6,39] because 0.25 mM ATP is already a saturating concentration. Increasing the ATP concentration to 4 mM does not increase the fraction of open clamps in assays with either of the Walker B mutants; and at all concentrations the relative intensity is lower than for WT RFC, about 1.2 to 1.3. If the weaker PCNA opening activity of the Walker B mutants were due to weaker ATP binding, the fraction of open clamps would be expected to increase with increasing ATP concentrations unless ATP concentrations of 4 mM are still not high enough for a significant fraction of RFC to bind ATP. 

To determine whether WB mutants are defective in ATP binding, ATP binding was measured more directly by using a fluorescent ATP analog, TNP-ATP. TNP-ATP is weakly fluorescent when free in solution, and the fluorescence increases when bound to a protein in a less polar environment [44,45]. Because the fluorophore is attached to ATP, the TNP-ATP concentration was held constant at 50 µM and the concentration of RFC was varied from 0.1 to 0.8 µM. For both WT RFC and WB mutants, TNP-ATP fluorescence increased linearly with increasing concentrations of RFC demonstrating that the WB mutants bind TNP-ATP as does WT RFC. Note that saturation in TNP-ATP binding was not observed due to the requirement of high concentrations of RFC (at least 10 µM) that were experimentally inaccessible. The small differences in TNP-ATP fluorescence between WT RFC and the WB mutants are not enough to account for the weak PCNA opening activity of the mutants given that the TNP-ATP concentration is much lower than the ATP concentrations used in Figure 4a.

Together, results in Figure 4 show that the RFC WB mutants bind ATP and that a lack of ATP binding cannot explain the weaker PCNA binding and opening activity of the WB mutants. Interestingly, in assays with no ATP (Figure 4a, 0 mM ATP), the same fraction of PCNA molecules is opened by the mutants as opened in assays with ATP suggesting that ATP does not stimulate PCNA opening by the WB mutants. Thus, mutation of the Walker B Glu residues negatively affects high affinity ATP-dependent PCNA binding and opening. A similar finding has been made for the conserved Arg finger residue that acts in trans to help catalyze ATP hydrolysis in the adjacent subunit. Mutation of the Arg finger motif in both the *E. coli* and *S. cerevisiae* clamp loaders does not affect ATP binding by the clamp loaders, but leads to defects in ATP-dependent DNA and clamp binding [46,47,48]. Taken together, these studies suggest that one simple function cannot be attributed to individual amino acid residues in the ATP active site; the contribution that these residues make to clamp loader function is more complex.

### 2.4. Measurement of DNA Binding

**Figure 5:**
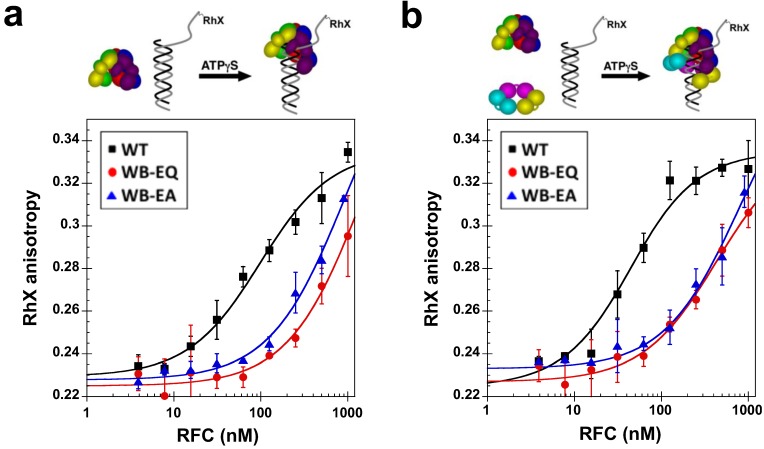
DNA binding activities of WT RFC and Walker B mutants. The anisotropy of RhX covalently attached to p/t DNA was measured as a function of RFC concentration. Binding of WT RFC (squares), the WB-EQ mutant (circles) and the WB-EA mutant (triangles) to DNA was measured in the presence of 0.5 mM ATPγS and 20 nM p/t DNA-RhX. (**a**) DNA binding was measured in the absence of PCNA. (**b**) DNA binding was measured in the presence of 2 µM PCNA. Three independent experiments were done and error bars represent the standard deviation.

DNA binding by RFC requires ATP binding, but not hydrolysis [5]. But, because the WB Glu mutations affect ATP-dependent interactions with PCNA, effects of these mutations on DNA binding were also measured. The binding of RFC to primed template (p/t) DNA was measured in an anisotropy-based assay. A 60-nucleotide template was labeled with X-rhodamine isothiocyanate (RhX) on the 5' end via an amino linker and annealed to a 26-nucleotide primer to create a p/t substrate with a 34-nucleotide 5'-template overhang. Assays contained 20 nM p/t DNA-RhX, 0.5 mM ATPγS and varied concentrations of RFC. ATPγS was used instead of ATP to inhibit DNA-triggered ATP hydrolysis and release of PCNA on DNA. Briefly, DNA and RFC were added sequentially to a cuvette containing buffer and ATPγS, and polarized intensities were measured after each addition (Figure 5a). The *K_d_* values calculated from RhX-aniostropy data for the clamp loaders binding DNA were 91 ± 28 nM (WT RFC), 690 ± 400 nM (WB-EQ) and 1,060 ± 200 nM (WB-EA). These values show that the WB mutations also decrease ATP-dependent DNA binding activities of the RFC mutants.

The DNA binding by RFC has been shown to be stimulated by PCNA binding [5]. Because our data shows that WB mutations decrease PCNA opening, we analyzed the effect of PCNA on DNA binding to determine whether PCNA would no longer stimulate DNA binding for the WB mutants. For these experiments, DNA binding was measured in RhX-anisotropy assays as above except that 2 µM PCNA was added to the cuvette after adding DNA and RFC, and the results are plotted for each RFC in Figure 5b. The *K_d_* values calculated for DNA binding in the presence of PCNA were 37 ± 13 nM (WT), 460 ± 200 nM (WB-EQ) and 1,800 ± 700 nM (WB-EA). Other studies showed that PCNA reduced the *K_d_* for RFC-DNA binding by about a factor of 3 [5], and this binding assay gave similar results for WT RFC. PCNA binding had no effect on DNA binding by the RFC WB mutants; binding was weak in both the absence and presence of PCNA. These data are consistent with expectations based on weaker PCNA binding and opening activities of the RFC WB mutants in that PCNA did not stimulate DNA binding by the mutants.

### 2.5. Measurement of ATP Hydrolysis

**Figure 6:**
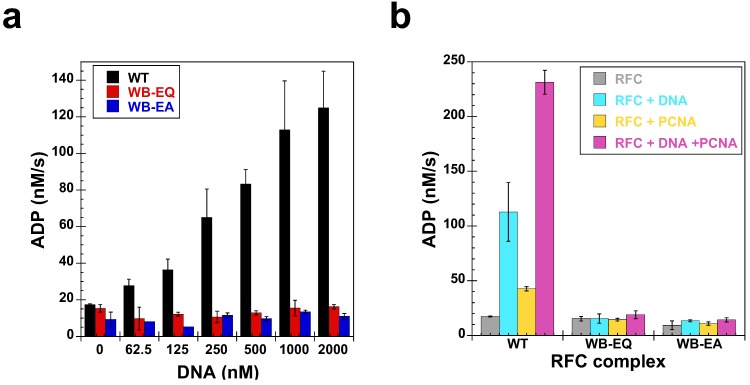
Effects of Walker B glutamate mutations on rates of ATP hydrolysis. (**a**) Rates of ATP hydrolysis were measured for WT RFC (black), the WB-EQ mutant (red) and the WB-EA mutant (blue) in the presence of 0.5 mM ATP and varying concentration of p/t DNA. The concentration of ATP hydrolyzed per second is plotted at several DNA concentrations for solutions containing 450 nM each RFC. (**b**) Rates of ATP hydrolysis were measured in assays containing 1 µM PCNA in the absence (yellow bars) and presence (magenta bars) of 1 µM DNA. Data from RFC alone (gray bars) and RFC with 1 µM DNA (cyan bars) are taken from panel a. Data shown are the average of three independent experiments. Error bars represent standard deviation.

The Walker B glutamate is predicted to activate a water molecule for catalysis of ATP hydrolysis [25,26,27]; therefore, weaker ATP hydrolysis activities are expected for the RFC WB mutants. ATPase activity was measured in a steady-state reaction that uses the coupled pyruvate kinase/lactate dehydrogenase assay to report on the ADP product produced. Briefly, pyruvate kinase uses the ADP product to convert phosphoenol pyruvate to pyruvate, and lactate dehydrogenase uses NADH to reduce pyruvate to lactate. Thus, one mole of NADH is converted to NAD^+^ per mole of ADP produced by hydrolysis. The fluorescence of NADH was monitored to measure ATP hydrolysis. To convert fluorescence units to concentration of ADP, a standard curve was generated by adding known amounts of ADP to the coupled enzyme system. Because RFC is a DNA-dependent ATPase, the concentration of DNA was varied in ATPase reactions (Figure 6a). Assays were done at *V_max_* conditions with respect to ATP concentration and contained 500 µM ATP which is 50-fold greater than the reported *K_m_* value of 9.7 ± 3 µM [49]. PCNA was not included in these assays. In the absence of DNA, steady-state hydrolysis rates were 17.4 ± 0.4 nMs^−1^ (WT), 15.3 ± 2.1 nMs^−1^ (WB-EQ) and 9.3 ± 3.9 (WB-EA) nMs^−1^. Hence, the basal rate of ATP hydrolysis was not affected by the WB mutations. On the other hand, the effects of the WB mutations on DNA-stimulated ATPase activity were large. At the highest concentration of DNA, 2 µM, ATP hydrolysis rates were 125 ± 20 nMs^−1^ (WT), 16 ± 1 nMs^−1^ (WB-EQ) and 11 ± 1 (WB-EA) nMs^−1^. While adding DNA stimulates ATPase activity of WT RFC by a factor of 7, DNA has no effect on ATPase activity of the RFC WB mutants. Given that the WB mutants bind DNA more weakly than WT RFC, decreased rates of ATP hydrolysis are expected at DNA concentrations where DNA binding to the WB mutants is low. However, based on the fractions of RFC bound at 2 µM DNA calculated from *K_d_* values obtained in Figure 5a, about 95% WT RFC should be bound to DNA, and about 75% of the RFC WB mutants should be bound to DNA. Therefore, the weaker DNA binding activity of RFC WB mutants (Figure 5a) cannot account for the lack of DNA-stimulation of ATPase activity at this high DNA concentration.

The ATPase activity of WT RFC is stimulated by PCNA to a lesser degree than by DNA, and stimulated to the greatest extent by the presence of both PCNA and DNA [50,51]. To determine whether PCNA would affect the ATPase activity of the Walker B mutants, ATPase activity was measured in assays containing 1 µM PCNA and 1 µM PCNA with 1 µM p/t DNA (Figure 6b). While PCNA alone increased the ATPase activity of WT RFC by a factor of about 2 and PCNA with DNA increased the ATPase activity of WT RFC by more than 10-fold, PCNA had no effect on the ATPase activities of the Walker B mutants. Under all conditions tested, the ATPase activity of the RFC WB mutants was about the same as the basal level of ATPase activity of WT RFC in the absence of PCNA and DNA.

## 3. Experimental Section

### 3.1. Buffers

Assay buffer contains 30 mM HEPES-NaOH, pH 7.5, 150 mM sodium chloride, 10 mM magnesium chloride, 0.5 mM EDTA, 2 mM DTT and 2% glycerol. Storage buffer for RFC contains 30 mM HEPES-NaOH, pH 7.5, 300 mM sodium chloride, 0.5 mM EDTA, 2 mM DTT and 10% glycerol. Storage buffer for PCNA is the same as RFC storage buffer except for the inclusion of 150 mM sodium chloride. RFC purification buffer A contains 30 mM HEPES-NaOH pH 7.5, 10% (v/v) glycerol, 0.5 mM EDTA, 1 mM DTT and 0.04% Bio-Lyte 3/10 ampholyte (Bio-Rad). RFC purification buffer B contains 30 mM HEPES-NaOH pH 7.5, 500 mM NaCl and 30 mM imidazole.

### 3.2. Construction of RFC Expression Vectors

RFC expression vectors generated previously include pLANT/RIL expressing Rfc1 and Rfc5 [37], pET-11 expressing Rfc2, Rfc3, and Rfc4 [37], and pET-11 expressing Rfc3 and Rfc4 [52]. The coding sequence for Rfc2 was PCR amplified from *S. cerevisiae* genomic DNA and subcloned into a pCDFDuet vector (Novagen) between NcoI and BamHI restriction sites in the first T7 expression cassette. In all *RFC1* constructs, the sequence encoding the first 283 amino acid residues was deleted and replaced with a sequence encoding a 6X His-tag and kinase recognition motif. Site-directed mutations were introduced into the coding sequences for individual RFC subunits using the QuikChange Mutagenesis kit (Strategene) as per the manufacturer’s instructions. Site-directed mutagenesis was done on vectors that contained coding sequences for only one RFC subunit, and mutant coding sequences were subcloned into expression vectors that contained multiple coding sequences. Vectors were co-transformed into *E. coli* BL21 (DE3) cells in the combinations listed in Table 1 to produce WT and mutant RFC complexes.

**Table 1:** Expression vectors used to produce wild-type RFC and Walker B mutants.

RFC Complex	Expression Vector	Coding Sequences
Wild-type RFC	pLANT/RIL	*RFC1* and *RFC5*
	pET-11	*RFC2*, *RFC3*, and *RFC4*
RFC WB-EQ	pLANT/RIL	*RFC1* (E425Q) and *RFC*5
	pET-11	*RFC3* (E118Q) and *RFC4*(E115Q)
	pCDFDuet	*RFC2* (E141Q)
RFC WB-EA	pLANT/RIL	*RFC1*(E425A) and *RFC*5
	pET-Duet1	*RFC3*(E118A) and *RFC4*(E115A)
	pCDFDuet	*RFC2*(E141A)

### 3.3. Proteins

WT and mutant RFC complexes were purified as described previously with modifications listed below [53]. Transformed *E. coli* BL21(DE3) cultures (2.4 L) were grown to an OD of 0.8 at 30 °C (Table 1). After cooling to 15 °C, IPTG was added to a final concentration of 1 mM and cells were incubated for an additional 18 h at 15 °C. Cells were harvested by centrifugation, resuspended in 30 mL Buffer A containing 1 M NaCl and lysed using a French press at 14,000 p.s.i. The lysate was clarified by centrifugation and diluted with buffer A to a final NaCl concentration of 150 mM. The diluted supernatant was applied to two 5 mL Hi-Trap SP columns (GE healthcare) attached in tandem and equilibrated with buffer A containing 150 mM NaCl. Protein was eluted in a 100-mL gradient of 150 to 600 mM NaCl. The five-subunit RFC complex eluted at approximately 450 mM NaCl. The fractions containing RFC complex were pooled and dialyzed overnight against buffer B. The dialyzed protein was applied to a 1-mL Hi-Trap chelating column (GE healthcare) charged with NiSO_4_ and equilibrated with buffer B. Protein was eluted using a 10-mL gradient of 30 to 500 mM imidazole. Fractions containing RFC eluted at approximately 450 mM imidazole. The fractions were pooled, dialyzed against RFC storage buffer and stored at −80 °C. Typical yields were 5–10 mg of RFC. PCNA was expressed, purified and labeled as previously described [42].

### 3.4. Oligonucleotides

A primed template consisting of a 26-nucleotide primer annealed to a 60-nucleotide template was used. The sequences are as follows: primer, 5'-ACA CGA CCA GTA ATA AAA GGG ACA TT; and template, 5'-TTC AGG TCA GAA GGG TTC TAT CTC TGT TGG CCA GAA TGT CCC TTT TAT TAC TGG TCG TGT. For anisotropy experiments, the 60-mer template was covalently labeled with X-rhodamine isothiocyanate (RhX) (Invitrogen) at by incorporation of a C6 amino linker on the 5'-end as described [54].

### 3.5. Equilibrium PCNA Binding and Opening Measurements

These fluorescence assays were done as described previously [43]. Data were fit to Equation 1 in which PCNA_o_ and RFC_o_ are the initial concentrations of PCNA and RFC, respectively, and I_max_ and I_min_ are the maximum and minimum fluorescence intensities, respectively, to calculate *K_d_* values.


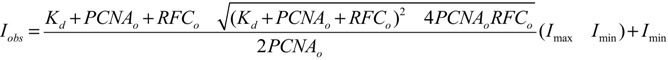
(1)

### 3.6. TNP-ATP Binding Measurements

The binding of 2',3'-O-(2,4,6-trinitrophenyl)-ATP (TNP-ATP) (Santa Cruz Biotechnology) to RFC was measured. TNP-ATP was excited at 408 nm and the emission spectra were recorded at 555 nm using a 3 nm bandpass. To generate each point, emission spectra were recorded after reagents were sequentially added to the cuvette. First, assay buffer was measured for a background signal, then 50 μM TNP-ATP for unbound ATP, and then RFC, from 0 to 0.8 μM was added to measure the signal for bound ATP. Storage buffer was added instead of RFC to generate the 0 nM RFC point. After correcting for buffer background, the intensity of bound ATP was divided by the intensity for free ATP for each point. All the values for 0 to 0.8 nM RFC were then divided by the value obtained for 0 nM RFC, setting this point to 1, to account for the change in fluorescence due to dilution.

### 3.7. DNA Binding Assay

The anisotropy of an RhX probe on the 26/60 mer p/t DNA was measured. RhX was excited at 585 nm and polarized emission was measured at 605 nm over 30 s using 8 nm bandpass as described [55]. The quadratic equation, (Equation 1) was used to calculate *K_d_* values for DNA binding except that the initial DNA concentration was substituted for the PCNA concentration, and maximum and minimum anisotropy values were substituted for intensity values.

### 3.8. ATP Hydrolysis Assay

ATPase data was collected on a QuantaMaster QM1 spectrofluorometer (Photon Technology International) at room temperature. NADH was excited at 340 nm and emission was measured at 460 nm using a 2 nm bandpass. To the assay buffer, 1 mM phosphoenolpyruvate, 0.2 mg/mL NADH, 68 units/mL pyruvate kinase, 99 units/mL lactate dehydrogenase, 0.5 mM ATP and varying concentrations of p/t DNA were added and mixed to give the final concentrations indicated. Reactions were initiated by adding 0.45 μM RFC (final concentration). Time courses were measured for 600 s and reaction rates were calculated from a linear fit of the time course data. Storage buffer was added instead of RFC to obtain the dilution effect on NADH fluorescence and a no enzyme reaction. To convert change in fluorescence to the rate of ADP produced, a standard curve was generated by adding varying concentrations of ADP (0 to 200 µM) to the coupled assay system and measuring the fluorescence at each ADP concentration. The slope obtained from a linear plot of fluorescence *vs*. ADP concentration was used to convert reaction time courses to nM ATP hydrolyzed/s.

## 4. Conclusions

Clamp loaders undergo a series of ligand-induced conformational changes that modulate the interactions of the clamp loader with the clamp and with DNA to allow the clamp loader to catalyze the mechanical clamp loading reaction. Two different residues, a conserved Asn (corresponding to N79 in Rfc4 [56]) and a conserved Arg (corresponding to R84 in Rfc4 [57]), have been proposed to function as “glutamate switches” to link ligand binding to ATP hydrolysis. In both cases, interaction between the switch residue and the DExx glutamate residue are proposed to position the Glu residue in a catalytically inactive conformation prior to DNA binding. The interaction between the switch residue and Glu changes when DNA binds to allow the Glu residue to adopt a catalytically active conformation. The loss of DNA-dependent, but not DNA-independent, ATPase activity by RFC Walker B mutants is consistent with, but does not prove, this model. However, our data may add another layer of complexity to the model. Because the mutation of the Glu residues decreases ATP-dependent ligand binding, perhaps interaction of the Glu residue with ATP possibly via a bridging water molecule helps position other residues to interact with PCNA and DNA. Then, interactions of these or other residues with PCNA and DNA reposition Glu to activate the water molecule for catalysis of ATP hydrolysis. When the Glu residue is altered, both ligand binding and ligand-induced ATP hydrolysis activities are reduced. It is interesting to note that while the Glu residue is conserved in the *E. coli* clamp loader DnaX subunits, the switch residues are not. Perhaps another residue functions as the switch residue in the *E. coli* clamp loader. Both this work and another study of the bacterial SecA protein suggest that the contribution that the Walker B Glu residue makes to the activity of mechanoenzymes is more complex than simply activating a water molecule for ATP hydrolysis [29].
